# Introduction of a Novel Swine-Origin Influenza A (H1N1) Virus into Milwaukee, Wisconsin in 2009

**DOI:** 10.3390/v1010072

**Published:** 2009-06-11

**Authors:** Swati Kumar, Michael J. Chusid, Rodney E. Willoughby, Peter L. Havens, Sue C. Kehl, Nathan A. Ledeboer, Shun-Hwa Li, Kelly J. Henrickson

**Affiliations:** 1 Midwest Respiratory Virus Program (MRVP) Laboratory, Medical College of Wisconsin, Milwaukee, WI 53226, USA; E-Mail: sxkumar@mcw.edu (S.K.); 2 Department of Pediatric, Medical College of Wisconsin, Milwaukee, WI 53226, USA; E-Mails: mchusid@mcw.edu (M.J.C.); rewillou@mcw.edu (R.E.W.); Phavens@mcw.edu (P.L.H.); kskehl@mcw.edu (S.C.K.); sli@mcw.edu (S.L.); 3 Department of Pathology, Medical College of Wisconsin, Milwaukee, WI 53226, USA; E-Mail: nledeboe@mcw.edu (N.A.L.); 4 Children’s Research Institute, Children’s Hospital of Wisconsin, P.O. Box 1997, Milwaukee, WI 53201-1997, USA; 5 Children’s Hospital of Wisconsin, P.O. Box 1997, Milwaukee, WI 53201-1997, USA; 6 Dynacare laboratories, Milwaukee, Wisconsin, USA

**Keywords:** S-OIV, novel influenza, outbreak, sub-typing, parainfluenza, respiratory viruses

## Abstract

On 17 April 2009, novel swine origin influenza A virus (S-OIV) cases appeared within the United States. Most influenza A diagnostic assays currently utilized in local clinical laboratories do not allow definitive subtype determination. Detailed subtype analysis of influenza A positive samples in our laboratory allowed early confirmation of a large outbreak of S-OIV in southeastern Wisconsin (SEW). The initial case of S-OIV in SEW was detected on 28 April 2009. All influenza A samples obtained during the 16 week period prior to 28 April 2009, and the first four weeks of the subsequent epidemic were sub typed. Four different multiplex assays were employed, utilizing real time PCR and end point PCR to fully subtype human and animal influenza viral components. Specific detection of S-OIV was developed within days. Data regarding patient demographics and other concurrently circulating viruses were analyzed. During the first four weeks of the epidemic, 679 of 3726 (18.2%) adults and children tested for influenza A were identified with S-OIV infection. Thirteen patients (0.34%) tested positive for seasonal human subtypes of influenza A during the first two weeks and none in the subsequent 2 weeks of the epidemic. Parainfluenza viruses were the most prevalent seasonal viral agents circulating during the epidemic (of those tested), with detection rates of 12% followed by influenza B and RSV at 1.9% and 0.9% respectively. S-OIV was confirmed on day 2 of instituting subtype testing and within 4 days of report of national cases of S-OIV. Novel surge capacity diagnostic infrastructure exists in many specialty and research laboratories around the world. The capacity for broader influenza A sub typing at the local laboratory level allows timely and accurate detection of novel strains as they emerge in the community, despite the presence of other circulating viruses producing identical illness. This is likely to become increasingly important given the need for appropriate subtype driven anti-viral therapy and the potential shortage of such medications in a large epidemic.

## Introduction

Beginning 17 April 2009, increased numbers of novel swine origin influenza A (H1N1) virus (S-OIV) cases began to appear in the United States [1]. As part of a rapid clinical and public health response, the Medical College of Wisconsin [Midwest Respiratory Virus Program (MRVP)] and the two affiliated academic private hospitals Children’s Hospital of Wisconsin (CHW) and Froedtert Hospital [Dynacare Laboratories (DL)] established full genetic subtyping of all influenza A viruses identified in patient samples sent to the respective clinical laboratories. We report information for the first 4 weeks of testing, during which 679 patient samples with S-OIV were identified in a Midwest American city.

Early detection of novel influenza A viruses of pandemic potential will be critical for minimizing the morbidity and mortality of such infections. Emergence of novel strains of influenza A can go undetected in the absence of the ability to subtype positive specimens or to differentiate currently circulating humans subtypes from emergent or reemergent novel subtypes [2,3,4]. The majority of influenza diagnostic assays currently utilized in clinical laboratories in the United States do not allow subtype determination.

The epidemiology of respiratory viruses in pediatric and adult populations during the 16 weeks preceding and first 4 weeks of the S-OIV outbreak demonstrates the sudden emergence of a novel virus causing a clinically indistinct influenza-like illness (ILI). This outbreak raises important diagnostic and therapeutic issues surrounding management of an influenza epidemic within the background of seasonal non-influenza respiratory virus activity. This is particularly important given the increasing need for subtype and antiviral susceptibility testing guided therapeutics for influenza and the shortage of antiviral medications [5].

## Results

Of 2678 children and adolescents (≤18 years) from whom respiratory specimens were collected from 26 April to 23 May 2009, 598 (weeks 17–20 of 2009) (22.3%) were positive for influenza A. Of these 598 children and adolescents with influenza A infections, 589 (98.5%) were identified as S-OIV, 9 (1.5%) human H1N1, and none were H3N2. Ninety-four of 1048 (8.9%) adults tested during the same time period were positive for influenza A, with 90 (95.7%) of influenza A positive adults having S-OIV, 2 (2.1 %) human H1N1 and 2 (2.1%) human H3N2.

The mean age of children and adolescents (≤18 years) and adults with influenza A was 8.2 years and 34.9 years respectively. The mean age of all patients with S-OIV infection was12.2 years and human H1N1 was 18.2 years (not significantly different, p=0.261). However, males aged 19–49 made up only 6.9% of all S-OIV in males while this age range made up 16.2% of S-OIV in females. Approximately 20% of males tested for influenza (N=1710) were positive for S-OIV in comparison to 16.5% of females tested for influenza (N=2016). Males represented 51% of all S-OIV detected (p=0.002). Analyses of percent of unique patients positive for S-OIV by age groups and gender is shown in Table 1.

The percent positivity rates were highest in school age children 5–11 year age group, both in males and females. The percent positivity rates were significantly higher for both the 5–11 year and the 12–18 year age groups than the age groups of < 2 years, 2–5 years, 19–49 years, 50–64 years and ≥ 65 years in the study (Table 1).

All S-OIV positive specimens were confirmed to be influenza A positive, swine H1 positive and not human H1 or H3 positive. In addition, 110 of the S-OIV positive clinical samples (100% of those tested) were shown to be swine N1 positive and human N1, N2, and H2 negative and negative to human/animal H5, H7, H9, N7 in subtyping assays. All 23/23 MRVP specimens tested at the Wisconsin State Laboratory of Hygiene (WI SLH) were confirmed to be correctly subtyped None of the 61 influenza A specimens collected from 22 January to 23 March 2009 were determined to be of swine origin.

**Figure 1.**
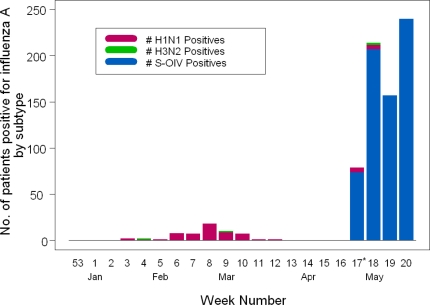
Influenza A virus activity over 2008–2009 winter and spring seasons by calendar week in Milwaukee, WI (U.S.). Patients positive for S-OIV and human influenza subtypes are depicted using bar charts. H1N1 and H3N2 are the seasonal human subtypes of influenza A. The * indicates the beginning of the S-OIV outbreak in Milwaukee, WI.

Subtype distribution of influenza A (in the populations from children and adults over the winter 2009 influenza season are displayed in Figure 1.

**Figure 2.**
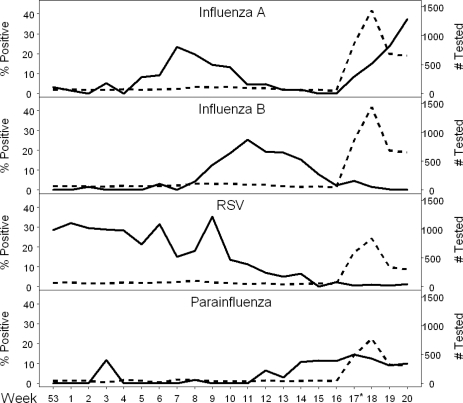
Respiratory virus activity over 2008–2009 winter season by calendar week. Total number of samples tested (as indicated by the dashed line and the righthand y-axis) and proportion positive (as indicated by the solid line and the lefthand y-axis) are depicted for 4 viruses by standard multiplex molecular assays for respiratory viruses. The * indicates the beginning of the S-OIV outbreak in Milwaukee, WI.

The local epidemiology of respiratory viruses circulating in the community (both children and adults) for the 16 weeks preceding the outbreak is shown in Figure 2. These data, obtained through weekly surveillance of respiratory viruses using molecular detection methods demonstrate that activity of non-S-OIV influenza A peaked from mid- February to mid- March and subsequently declined to a minimal level with no cases detected in the two weeks preceding the onset of detection of S-OIV. At the time of onset of the S-OIV outbreak (week 17 of 2009), parainfluenza was the major respiratory virus (of those tested) circulating in the community (maximum 11.1% weekly positivity rate) along with declining levels of influenza B and RSV at 2.4 % and 2.3 % respectively. Human coronaviruses (HCoVs) and rhinoviruses (HRVs) were not tested during this out break. A small number of CHW samples were tested by PCR for adenovirus (# positive / # tested): 7/47 (14.9%) week 17, 7/39 (17.9%) week 18, 0/7 (0%) week 19, 1/18 (5.6%) week 20, 1/16 (6.3%) week 21 of 2009. It is probable that a significant percentage of the samples that tested negative for influenza A, B, RSV, HPIV 1, 2, 3, ADV, and S-OIV would test positive to HCoV or HRV. Influenza B accounted for all of the influenza strains detected in the 2 weeks preceding the outbreak. Enhanced surveillance conducted subsequent to the first positive case of S-OIV demonstrated a sharp spike in influenza A activity from no isolates to N=79 (8.3% positive of those tested) in Week 1, a small transient increase in influenza B activity (N=44; 4.6% positive), and co-circulation of parainfluenza viruses with detection rates of the latter (14.4%) exceeding that of S-OIV during the first week of the outbreak (week 17 of 2009) (Figure 2). Influenza A activity continued to rise during the next 3 weeks of surveillance with detection rates in patients tested of 14.9%, 23.7% and 37.5% per week respectively (Duplicate clinical samples were removed before data analysis). Influenza B and RSV continued to be detected at low levels with weekly detection rates ranging from 0.2–1.7% and 0.6–1.3% for the two viruses respectively. Parainfluenza remained the most prevalent seasonal respiratory viral agent with detection rates of 8.9–12.6% in the last 3 weeks of study period.

**Figure 3.**
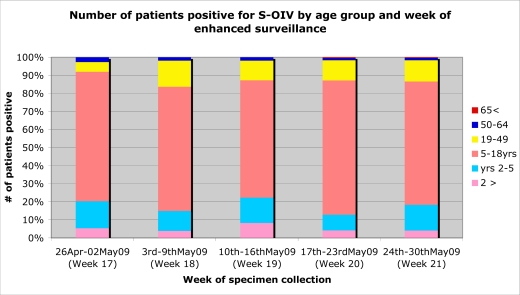
Age distribution of S-OIV positive patients by week of enhanced surveillance.

**Figure 4.**
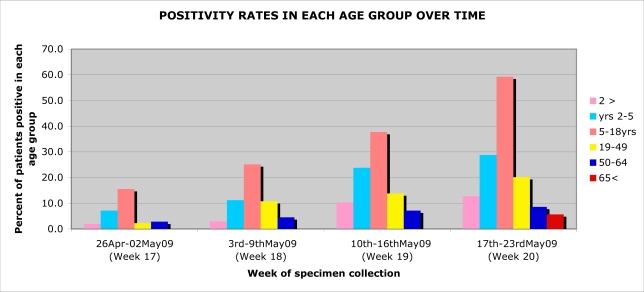
Percent patient positivity by age group and week of enhanced surveillance.

Seasonal human subtypes of influenza virus continued to be detected at low levels with daily patient positivity rates of 0%–2.5% during the first two weeks of the study period (weeks 17–18 of 2009), versus S-OIV daily positivity rates of 5.8%–58.2% in children and 0–3.7 % and 0%–40% respectively in adults. S-OIV percent positivity and total number of infections were significantly greater in children compared to adults during the first four weeks of the outbreak (weeks 17–20 of 2009) (Table 1, Figure 3). Of note, only 6 adults (2.4% of those tested) with S-OIV were identified in comparison to 68 (9.6 % of those tested) children in the first week of enhanced surveillance. The rate of positive tests for children and adults increased dramatically over the next 3 weeks (Figure 4). This most likely reflects the change in testing recommendations during the second week of May to test only seriously ill or high risk patients. Interestingly the number of patients between 5–18 that were tested each week remained fairly stable except for the first week (n=53,143,102, 179).

**Figure 5.**
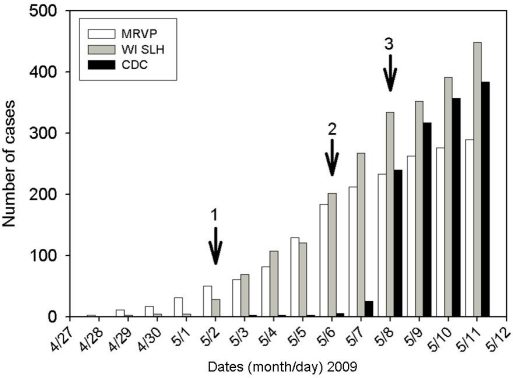
Cases of S-OIV reported in Wisconsin as enumerated by MRVP, WI SLH and CDC surveillance by calendar day during first 2 weeks of the S-OIV epidemic. Arrow 1: Cases reported by MRVP were validated to be “probable” S-OIV by WI SLH. MRVP cases were then included automatically in SLH totals. Arrow 2: Cases reported by MRVP were validated as “confirmed” by WI SLH using the CDC assay. MRVP and SLH cases were then included automatically in CDC totals. Arrow 3: testing is restricted to high risk patients. Note that the MRVP samples only come from southeastern Wisconsin, while the WI SLH cases include reporting from throughout the state.

The number of cumulative cases in Wisconsin being independently reported by the CDC, the WI SLH and the MRVP during the first two weeks of the outbreak can be seen in Figure 5. The number of cases identified at our laboratory exceeded the number reported by the WI SLH for the first 5 days of the epidemic. Rapid validation of our in-house subtyping assay by WI SLH enabled subsequent timely reporting of the number of cases by the WI SLH as early as the 6^th^ day of the epidemic.

## Discussion

Availability of molecular assays that rapidly and accurately detect and differentiate simultaneously circulating subtypes of influenza A allowed us to confidently detect S-OIV infections in our community within a few days of the first nationally reported cases. Timely detection of the first case was critical for alerting public health officials to the presence of the novel influenza strain in the region. Subsequent rapid screening of patients with ILI symptoms, and subtyping of all influenza A positive samples was instrumental in identifying and assessing the evolution and extent of the outbreak in a timely manner. This provided information critically needed by both local and state public health officials for making decisions regarding utilization of diagnostic and therapeutic resources and public health interventions.

Retrospective testing of influenza A positive specimens collected from the Southeastern WI region prior to the onset of S-OIV epidemic suggested that the outbreak was detected shortly after entry into the community. This was strongly evidenced by the absence of any influenza A positive specimens for 2 weeks prior to 28 April 2009 as well as the absence of S-OIV detection in all 61 specimens available for retrospective subtype testing going back to 22 January 2009.

All subtypes of seasonal human influenza were found during the outbreak period at low, but detectable levels. Continued detection of seasonal human influenza during the S-OIV outbreak demonstrates the persistent presence of these viruses after their apparent “disappearance” from a community. The enhanced respiratory virus testing stimulated by the S-OIV epidemic led to increased identification of seasonal human influenza isolates in clinical samples. Utilization of multiplex assays for respiratory virus diagnostics, coupled with routine subtyping of influenza A positive samples, is important to identify respiratory disease due to an unusually long persistence of seasonal influenza or emergence of novel strains of the virus. Post -seasonal influenza outbreaks caused by clinically indistinct influenza strains could otherwise be obscured by seasonal surges from concurrently circulating parainfluenza or other respiratory viruses in the spring. Therefore, it is possible that novel strains of influenza have emerged previously and been unrecognized because they caused relatively mild disease and were not identified.

The apparent lower rates of S-OIV infection in adults throughout the study period and delayed time to peak positivity compared to children suggest that pediatric surveillance may be a more sensitive tool for early detection of novel influenza strains entering in the community. Greater numbers of children were being tested on any given day than adults despite identical screening criteria for both populations.

The WI SLH validation of the subtyping assays developed in our laboratory within 5 days of the report of the first positive specimen at our site demonstrates the close collaboration between the two laboratories, in accordance with the provisions of the Laboratory Response Network (LRN) established by the CDC. Rapid incorporation of private clinical or research diagnostic laboratories with pre-existing resources and expertise for molecular diagnostic testing into the LRN or a separate emergency network accelerates the early detection of novel pathogens in local communities and provides the diagnostic laboratory “surge capacity” required during an outbreak or pandemic. Detection and evolution of an outbreak in real time is important for rapid implementation of community mitigation measures such as school closures, that have been suggested to be important for reducing transmission and subsequent morbidity and mortality in an outbreak [6,7]. Mathematical modeling of the 1918 pandemic showed that public health interventions to reduce influenza transmission were efficacious only when implemented at very early stages of an epidemic [8].

Contemporaneous circulation of seasonal and novel influenza A viruses illustrates the increasing role and importance of subtyping in providing appropriate patient care. The seasonal human H1N1 circulating during the 2008–2009 influenza season was resistant to oseltamivir but sensitive to adamantanes while the seasonal H3N2 subtype and S-OIV strains were sensitive to oseltamivir and resistant to adamantanes [9]. Empiric use of oseltamivir was complicated by co-circulation of other influenza viruses and equal amounts of parainfluenza virus. At a time when pharmacies were depleted of oseltamivir in our city, between 6 and 10 patients needed to be treated empirically for each actual S-OIV infection we identified. Subtype driven influenza diagnostic testing using rapid assays would help preserve and direct appropriate utilization of limited antiviral reserves in an outbreak.

## Method

Routine detection of influenza A at CHW and DL clinical laboratories is accomplished by a multiplex real-time reverse transcription polymerase chain reaction (RT-PCR) assay for detection of influenza A, influenza B and respiratory syncytial virus (RSV) that does not subtype influenza A [10]. CHW and DL laboratories send positive specimens to the MRVP for influenza subtyping. The MRVP employs up to 5 different multiplex assays utilizing real time PCR and end point PCR that fully subtype both human and animal influenza viruses [11,12]. In addition, it performed specific detection of S-OIV and sequencing of this virus. These assays are rapid and results, run 4–6 times daily, were often reported within 3–7 hours. Nineteen S-OIV and 4 human H1N1 specimens subtyped at the MRVP were subsequently independently tested and confirmed by the WI SLH using the CDC influenza typing and subtyping assays and S-OIV specific detection kit [13].

Within 4 days of the report of the first 8 cases of S-OIV infections in the United States by the Centers of Disease control [CDC, 14] routine subtype determination commenced at the MRVP. On 28 April 2009 (day 2 of subtype testing), three of 5 influenza A positive specimens were unable to be subtyped as human H1 or H3 using a real time multiplex typing and subtyping assay developed to detect and differentiate seasonal H1, H3, and potentially pandemic H5, H7, and H9 subtypes of influenza A [11]. Further characterization of these unsubtypable influenza A isolates was accomplished with a 12-plex influenza subtyping RT-PCR enzyme hybridization assay (EHA) that detects and differentiates all influenza A subtypes known to infect humans including the N1 animal gene of S-OIV [12].

Subsequent to the confirmation of the first S-OIV positive specimen, active surveillance for S-OIV was expanded to all patients with an ILI in accordance with the broad guidelines proposed by the CDC for testing all ‘suspected cases’ at the onset of the outbreak [14]. Influenza-like-illness (ILI) was defined as fever (temperature of 100°F [37.8°C] or greater) and a cough and/or a sore throat in the absence of a known cause other than influenza [15]. Later, on 8 May 2009, the screening criteria were changed to become more restrictive, prioritizing testing patients with moderate to severe ILI or those with ILI with risk factors for complications of S-OIV infection. Moderate –severe ILI was defined as ILI with evidence of lower respiratory involvement (increased respiratory rates for age with or without accessory muscle use) but not requiring supplemental oxygen, hemodynamic or ventilator support. Patients considered to be at high risk for S-OIV complications were the same age and risk groups as those at higher risk for seasonal influenza complications (< 5 years or ≥ 65 years of age, children and adolescents who are receiving long-term aspirin therapy, pregnant women, patients with chronic medical conditions, patients with imunosuppression or residents of nursing homes and other chroniccare facilities) [16]. Testing criteria were further modified by local health authorities on 11 May 2009, to recommend testing of only those who were severely ill (e.g., hospitalized or had symptoms of LRI requiring hemodynamic or respiratory support) or who belonged to the high risk groups. Respiratory specimens tested during the outbreak were obtained from both hospitalized and ambulatory patients of all ages from Southeastern Wisconsin region. The majority of pediatric specimens tested in the study originated from non-hospitalized children, however the precise distribution of adults between inpatient and outpatient groups was not known. All samples received in the laboratory were processed as previously described [17].

Prospectively collected respiratory virus epidemiology and patient demographic data for the same populations for the 4 months preceding the outbreak were analyzed. For patients with duplicate samples only the earliest dated sample is included in this analysis. All 61 frozen influenza A positive clinical specimens available from the 81 samples collected between 22 January and 23 March 2009 were retrospectively subtyped to identify the date of S-OIV introduction into the Milwaukee area.

The definition of a “confirmed” case includes specimens determined to be positive for S-OIV at the CDC or using molecular assays developed and distributed by the CDC for S-OIV detection or assays validated for S-OIV detection with the CDC kit as the gold standard. Early in the outbreak many samples were determined to be “probable” by the WI SLH pending CDC confirmation; all have now been confirmed as S-OIV.

All data were collected under a protocol approved by the Institutional Review Board of Children’s Hospital of Wisconsin and the Medical College of Wisconsin.

## Conclusions

Reliance on nationally reported case numbers for determining the presence or absence of novel agents during epidemics and extent of disease in a given region is certain to be misleading given the inherent delays involved in ‘official’ confirmation, tabulating and eventual reporting of “confirmed” cases (Figure 5). The presence of a molecular laboratory that had already developed tests for rapidly subtyping influenza A viruses allowed for the rapid and early characterization of one of the largest local outbreaks of a novel influenza A virus in the United States. Continued local surveillance by such laboratories should help define important epidemiologic and virologic characteristics of these viruses and help facilitate future public health responses. Co-circulation of all three subtypes during the next influenza season will likely require routine determination of seasonal and novel influenza subtypes in order to guide therapeutic decisions for infected patients.

## Figures and Tables

**Table 1.** Percent positivity rates of S-OIV by age group and gender.

Males*	**< 2 yrs**	**2–5 yrs**	**5–11 yrs**	**12–18 yrs**	**19–49 yrs**	**50–64 yrs**	**>=65 yrs**	**Total**

# Patients positive	16	38	177	86	24	5	1	347
% Patients positive of positive patients	4.61	10.95	51.01	24.78	6.92	1.44	0.29	100%

# Patients tested	**372**	**276**	**474**	**261**	**203**	**75**	**49**	**1710**

% Patients positive of patients tested	**4.3**	**13.8**	**37.3**	**33.0**	**11.8**	**6.7**	**2.0**	**20.3**



Females**	**< 2Yrs**	**2–5 yrs**	**5–11 yrs**	**12–18 yrs**	**19–49 yrs**	**50–64 yrs**	**>=65 yrs**	**Total**

# Patients positive	18	39	134	81	54	7	0	333
% Patients positive of positive patients	5.41	11.71	40.24	24.32	16.22	2.10	0.00	100%

# Patients tested	**318**	**249**	**430**	**317**	**511**	**139**	**52**	**2016**

% Patients positive of patients tested	**5.7**	**15.7**	**31.2**	**25.6**	**10.6**	**5.0**	**0.0**	**16.5**

								

**TOTAL**	**34**	**77**	**311**	**167**	**78**	**12**	**1**	**680**

**% of S-OIV**	4.93	14.68	34.38	28.89	10.92	5.61	0.99	100%

* Chi-square=204.85; Degree of Freedom=6,

** Chi-square=149.659, Degree of Freedom=6,

The numbers of positive and negatives are significantly associated with age group for each gender (P<0.00001).
